# Supplementary figures and images for: Overexpression of MEKK18 from Arabidopsis pumila in rice significantly enhances stress resistance at the early stage
Source: PLoS One. 2025 Jun 25;20(6):e0325550. doi: 10.1371/journal.pone.0325550 (PMC12194022; doi:10.1371/journal.pone.0325550)

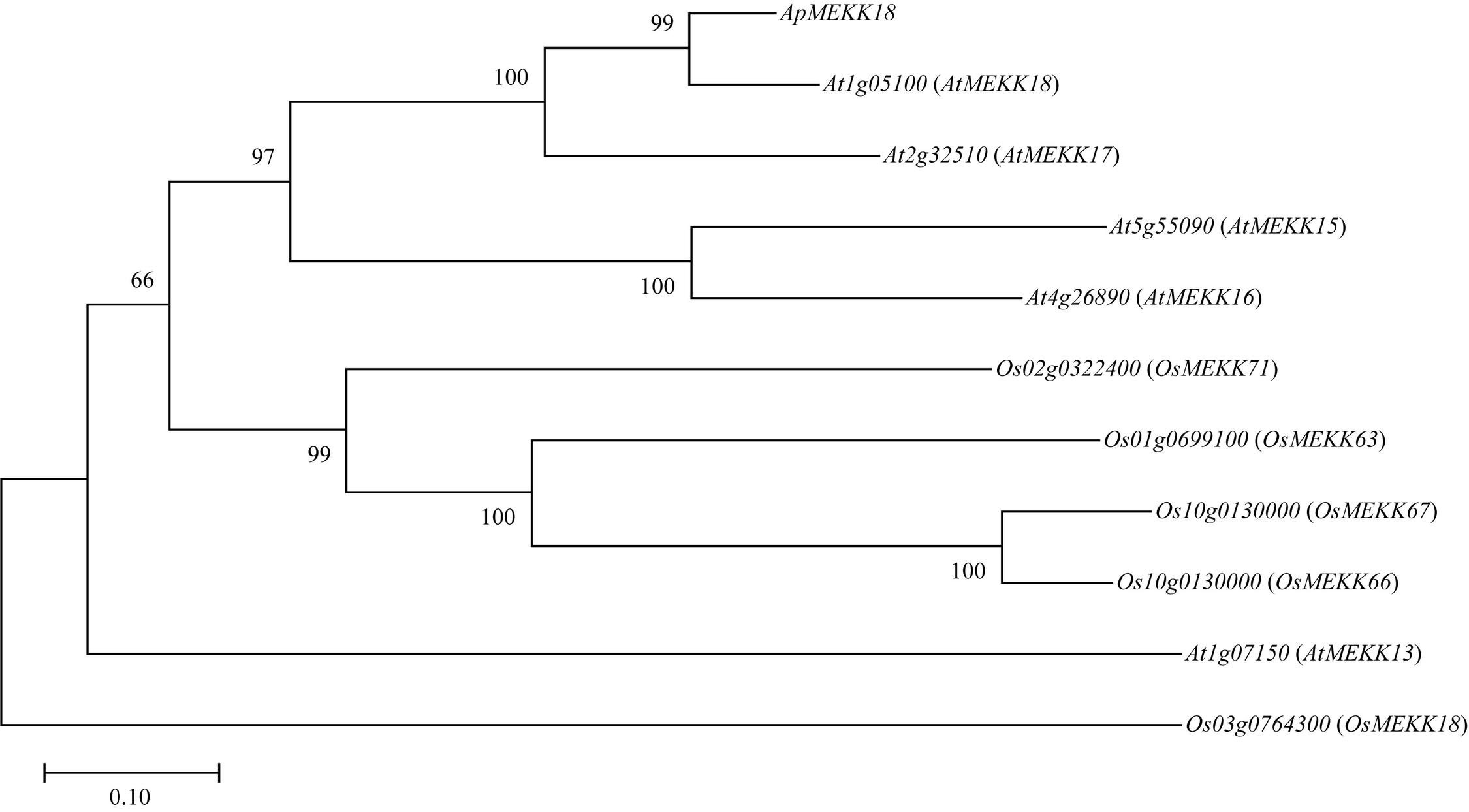

Supplement: S1 Fig — (TIF) [file pone.0325550.s001.tif]

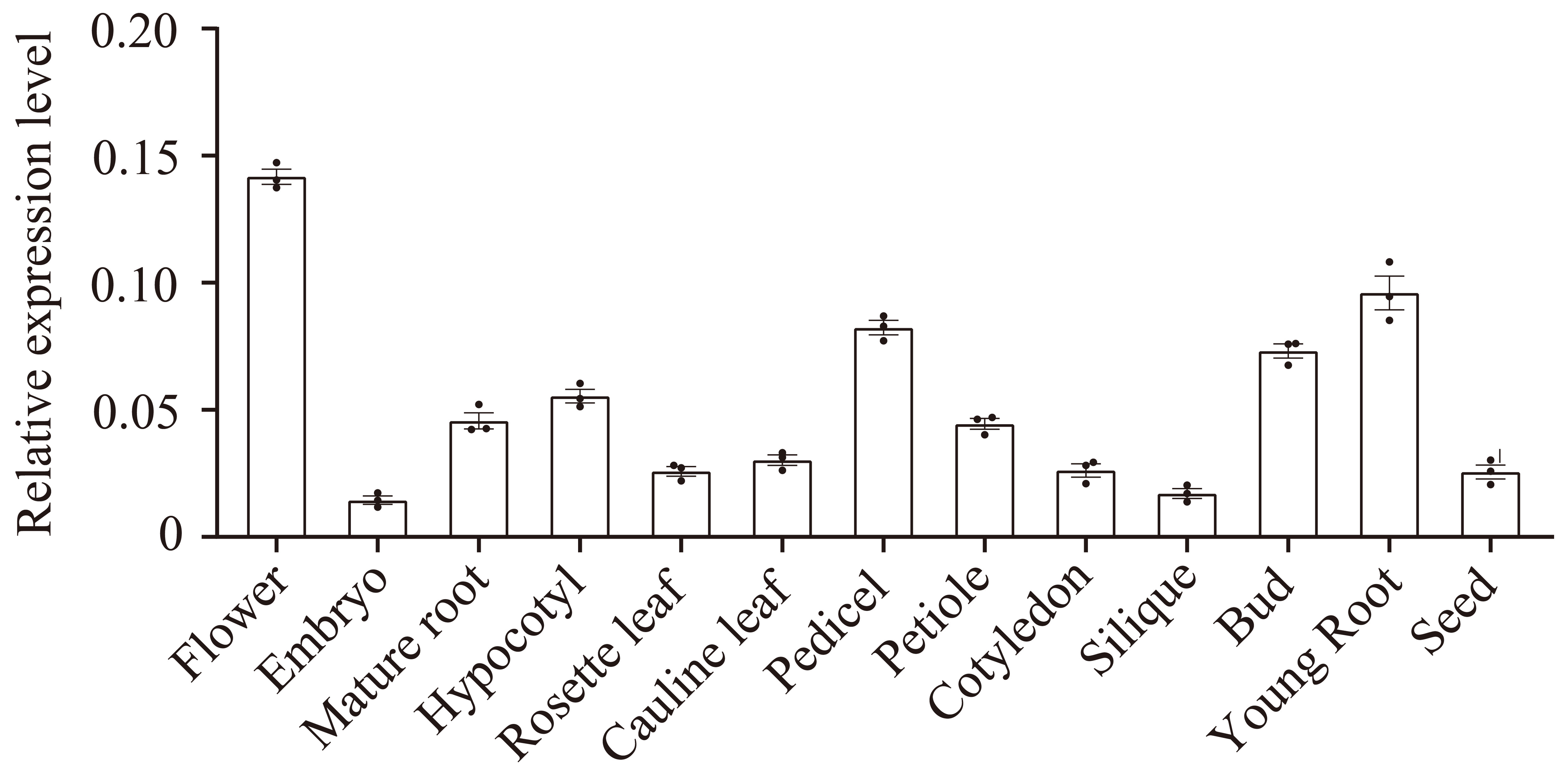

Supplement: S2 Fig — (TIF) [file pone.0325550.s002.tif]

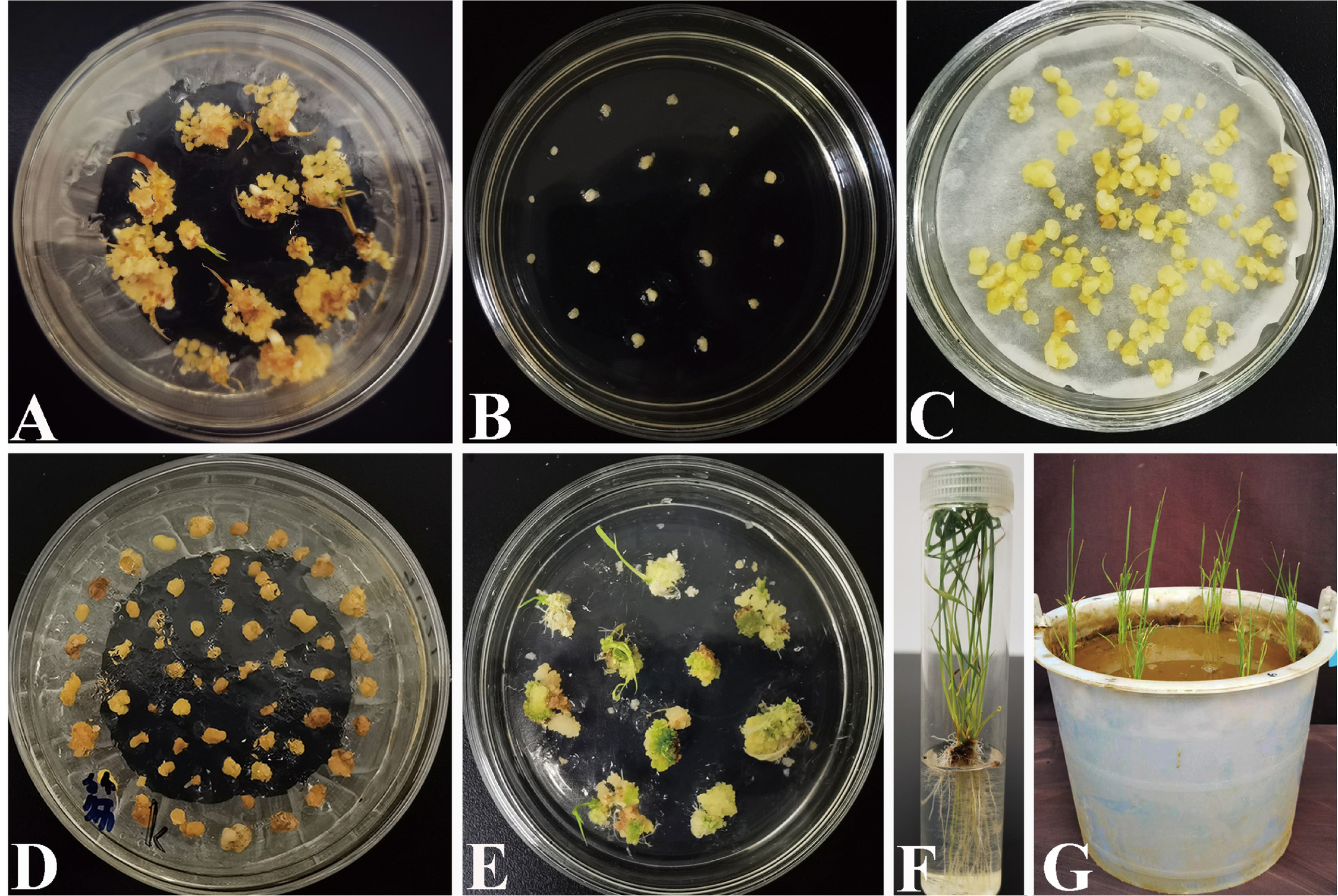

Supplement: S3 Fig — (A) Inducing callus tissue; (B) Succession cultivation; (C) Cocultivation; (D) Choose cultivation; (E) Differentiation cultivation; (F) Rooting; (G) Transplant. (TIF) [file pone.0325550.s003.tif]

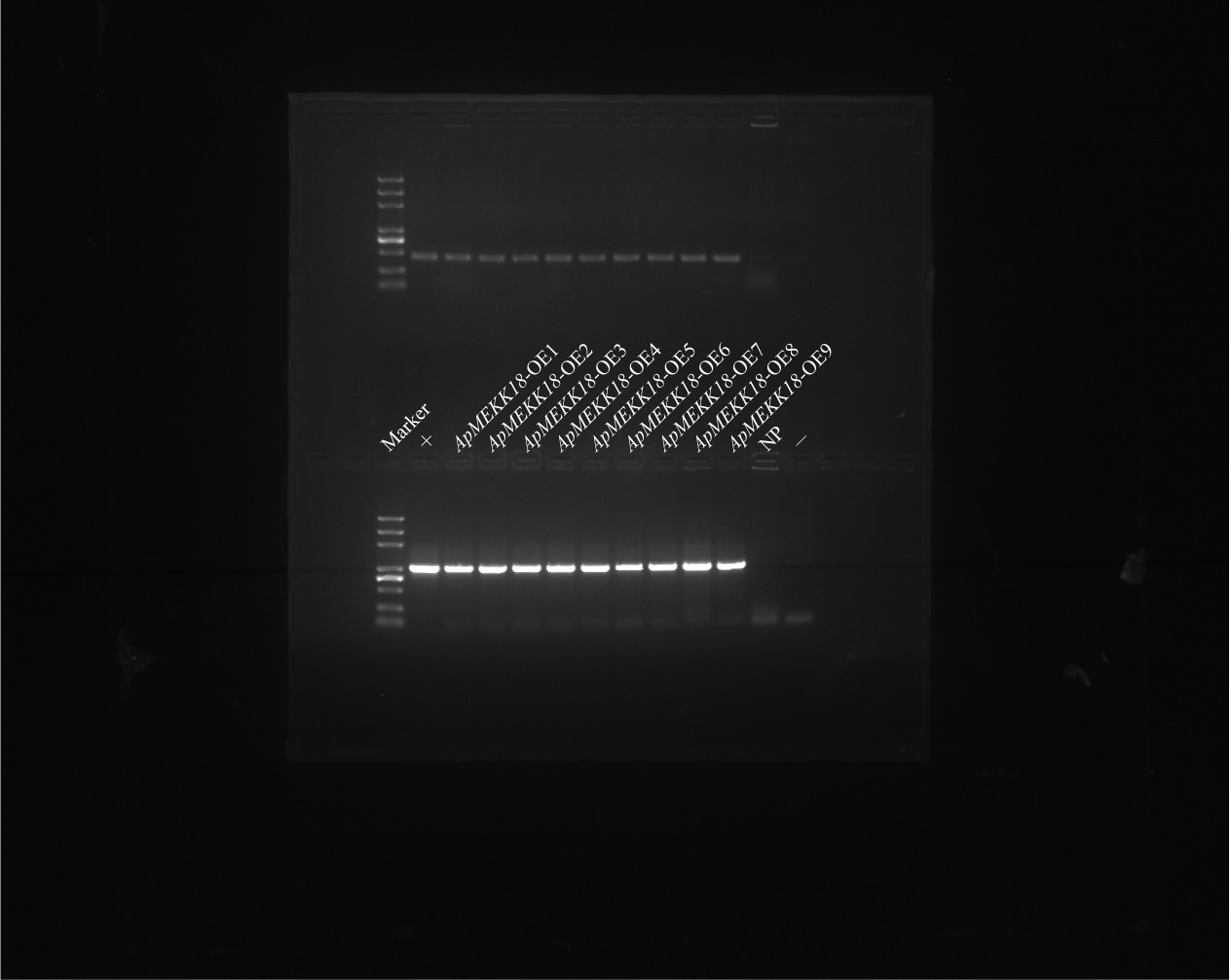

Supplement: S4 Fig — Maker: DL5000; + : Positive control; -: Negative control; NP: Nipponbare; OE-1 ~ 9 represent nine independent ApMEKK18 transgenic rice lines. The upper half of the electrophoresis gel was not related to this study. (TIF) [file pone.0325550.s004.tif]

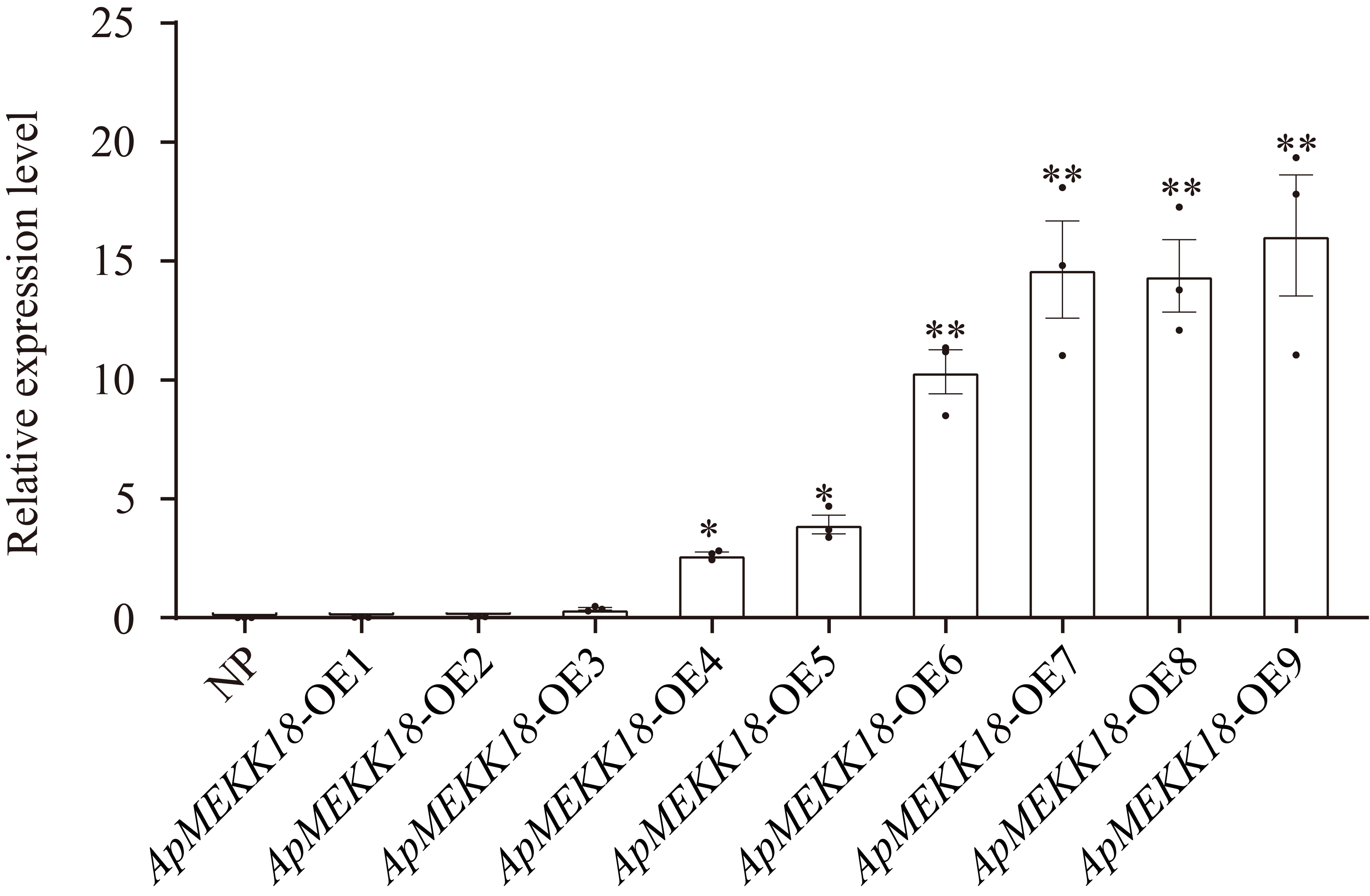

Supplement: S5 Fig — NP: Nipponbare; OE-1 ~ 9 represent independent ApMEKK18 transgenic rice lines; one-way ANOVA (Student’s t-test), *differences were significant (P < 0.05), **differences were highly significant (P < 0.01). (TIF) [file pone.0325550.s005.tif]

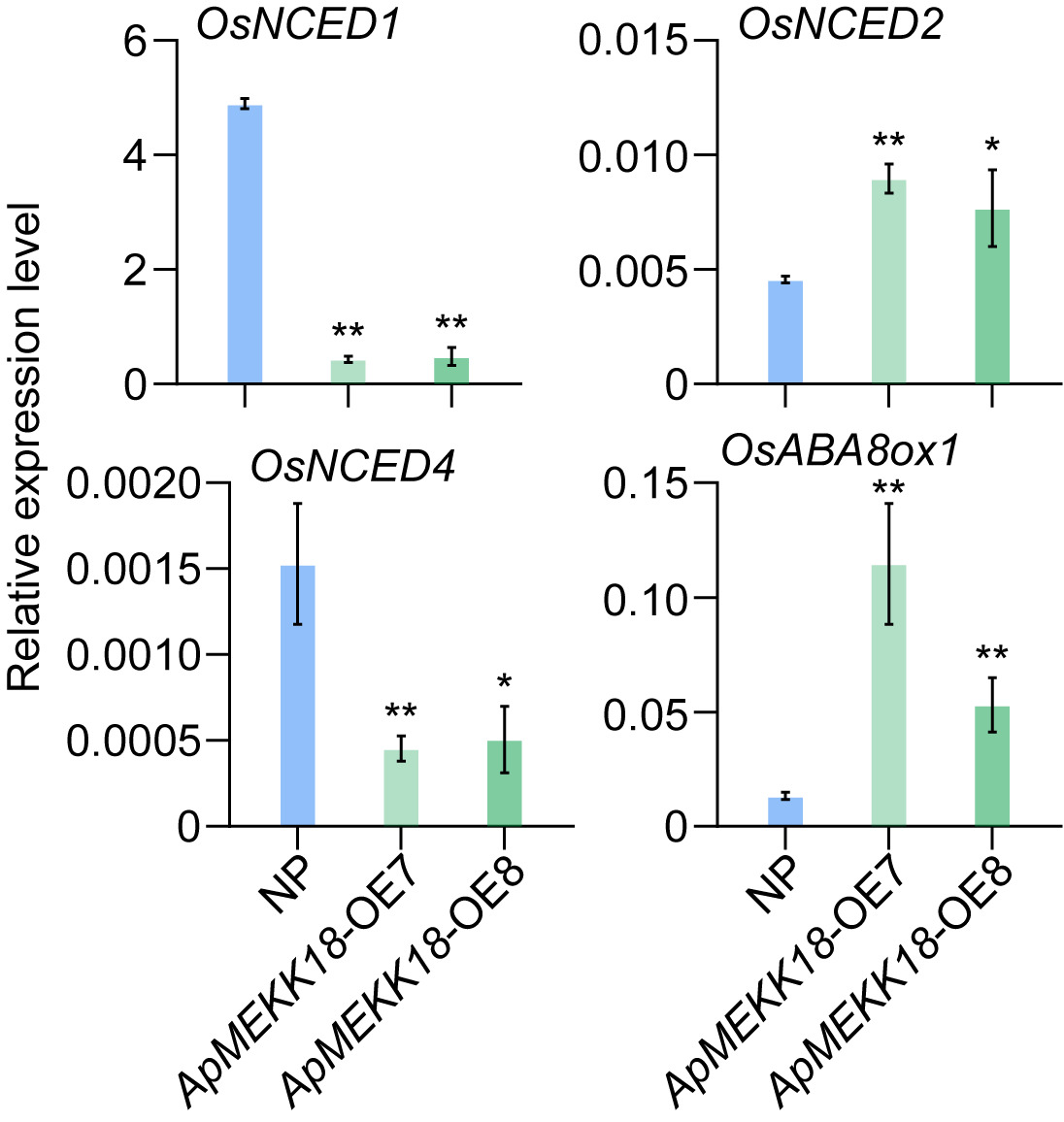

Supplement: S6 Fig — OsNCED1, OsNCED2, and OsNCED4: 9-Cis-Epoxycarotenoid Dioxygenase; OsABA8ox1, ABA 8′-Hydroxylase. *differences were significant (P < 0.05), **differences were highly significant (P < 0.01). (TIF) [file pone.0325550.s006.tif]

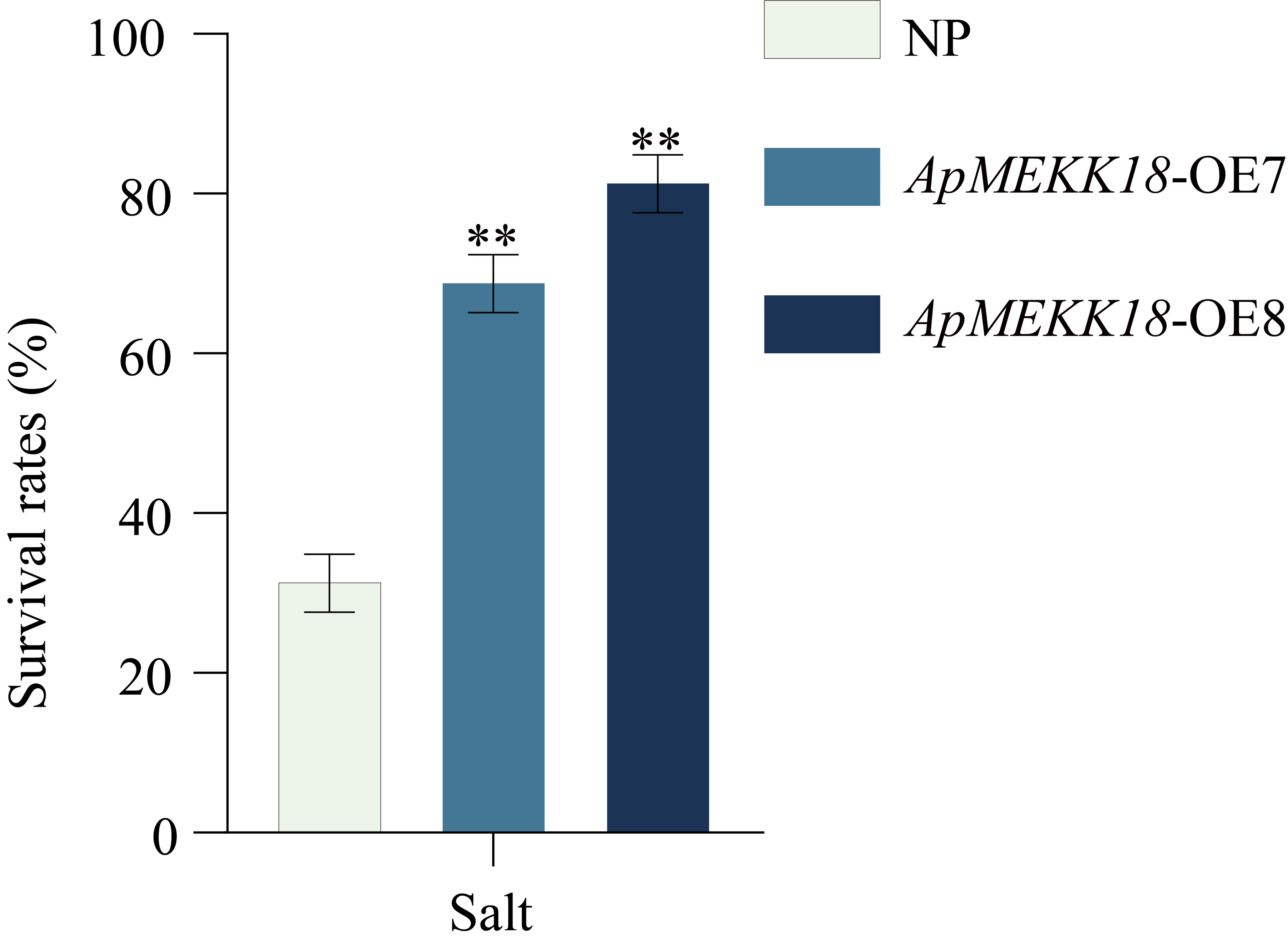

Supplement: S7 Fig — NP: Nipponbare; OE-7 and OE-8 represent two independent ApMEKK18 transgenic rice lines; one-way ANOVA (Student’s t-test), * P < 0.05, ** P < 0.01. (TIF) [file pone.0325550.s007.tif]
