# Supplementary material for: Overexpression of MEKK18 from Arabidopsis pumila in rice significantly enhances stress resistance at the early stage
Source: PLoS One. 2025 Jun 25;20(6):e0325550. doi: 10.1371/journal.pone.0325550 (PMC12194022; doi:10.1371/journal.pone.0325550)
Supplement: Attachment — Submitted filename: Response_to_reviewers_auresp_2.docx [file pone.0325550.s012.docx]

**Dear editors,**

Thank you very much for taking the time to review our manuscript entitled “**Overexpression of *MEKK18* from *Arabidopsis pumila* in rice significantly enhances stress resistance**” (Submission ID: PONE-D-25-02616) again.

According to the comments of Reviewer #2, we have made a careful correction in the revised manuscript. We hope this meets the requirements for a publication. All the revised sections of the text were highlighted using track changes. Our specific responses to each question are as follows:

**We ensure that our references are complete and correct, and we have not** **cited papers that have been retracted.**

**Reviewer #2:** The authors addressed all of my comments and suggestions with additional experimental data, which improves and clarifies the manuscript. However, a couple of points should be further considered. Fig. S6 showing the expression levels of ABA-related genes is crucial for supporting the role of ApMEKK18 in ABA response. First, the description in line 368-369 “while ABA 8′-Hydroxylase (OsABA8ox1) was significantly down regulated in ApMEKK18-OE8 (S6 Fig)” is wrong; its level was upregulated in the transgenic lines. Moreover, it is necessary to analyze the expression levels of these genes in other transgenic line (OE 7 line) to further support the conclusion. In addition, it should be discussed in Discussion section that how the up- and down-regulation of these genes is associated with the ABA-responsive phenotype of the plants.

**Response: Thank you for your careful reviews. We apologized for the mistake and corrected it in the revised manuscript. We have analyzed the expression levels of these genes in *ApMEKK18-OE7* line, and revised the S6 Fig. in the revised manuscript.** **Furthermore, we added the corresponding descriptions of the implications of changes in gene expression levels in Discussion section.**
